# 1063. ARGONAUT-V: Susceptibility of Multidrug-Resistant (MDR) *Pseudomonas aeruginosa* to Cefepime-Taniborbactam

**DOI:** 10.1093/ofid/ofab466.1257

**Published:** 2021-12-04

**Authors:** Andrew R Mack, Christopher Bethel, Steven Marshall, Robin Patel, Robin Patel, David van Duin, Vance G Fowler, Daniel D Rhoads, Michael Jacobs, Focco van den Akker, David A Six, Greg Moeck, Krisztina M Papp-Wallace, Robert A Bonomo

**Affiliations:** 1 Case Western Reserve University & Louis Stokes Cleveland VA Medical Center, Cleveland, Ohio; 2 Louis Sokes Cleveland VA Medical Center, Cleveland, OH; 3 Louis Stokes Cleveland Medical Center, Cleveland, OH; 4 Mayo Clinic, Rochester, MN; 5 University of North Carolina, Chapel Hill, North Carolina; 6 Duke University, Durham, North Carolina; 7 Cleveland Clinic, Cleveland, Ohio; 8 University Hospital Cleveland Medical Center, Cleveland, OH; 9 Case Western Reserve University, Cleveland, Ohio; 10 Venatorx Pharmaceuticals, Inc., Malvern, Pennsylvania; 11 Venatorx Pharmaceuticals, Malvern, Pennsylvania; 12 Louis Stokes Cleveland VAMC and Case Western Reserve University, Cleveland, OH; 13 Louis Stokes Cleveland VA Medical Center, Cleveland, OH

## Abstract

**Background:**

*P. aeruginosa* is a Gram-negative pathogen responsible for many serious infections. MDR, both intrinsic and acquired, presents major clinical challenges. Taniborbactam (formerly VNRX-5133; Fig 1) is a β-lactamase inhibitor (BLI) characterized as a bicyclic boronate, uniquely possessing activity toward all four Ambler classes of β-lactamases, both serine and metallo, with the exception of class B IMP β-lactamases. The β-lactam-BLI (BL-BLI) combination cefepime-taniborbactam (FTB; Fig 1) is currently in phase 3 clinical trials.

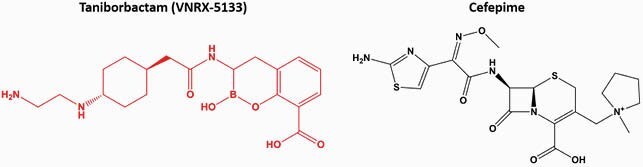

Structures of taniborbactam and cefepime. The β-lactamase inhibitor is in red and the β-lactam antibiotic is in black.

**Methods:**

The activity of FTB was tested against 197 well-characterized clinical *P. aeruginosa* isolates that were part of PRIMERS (Platforms for Rapid Identification of MDR-Gram-negative bacteria and Evaluation of Resistance Studies). Nearly 58% of these strains were reported as carbapenem-non-susceptible. Porin changes, efflux pumps, and/or the presence of acquired class A or class B carbapenemases were previously reported. Broth microdilution minimum inhibitory concentrations (MICs) were determined by CLSI M07 Ed. 11 methods with custom Sensititre frozen panels and interpreted using CLSI M100 Ed. 30 breakpoints. American Type Culture Collection strains were used for quality control. FEP breakpoints were provisionally used for FTB, where taniborbactam was fixed at 4 µg/mL.

**Results:**

Percent susceptibility to BL agents alone was 45.2% for imipenem (IPM), 55.8% for meropenem (MEM), 60.9% for ceftazidime (CAZ), and 67.0% for FEP. The addition of BLI to BL increased % susceptibility for MEM-vaborbactam (MVB), 56.9%; ceftolozane-tazobactam (C/T), 77.7%, CAZ-avibactam (CZA), 79.7%, and FTB, 82.7%. MIC_50_s were in the susceptible range for all drugs except IPM, which was intermediate, and all MIC_90_s were in the resistant range (Table 1). Taniborbactam reduced FEP MIC by 2-fold in 32% of isolates and ≥ 4-fold in 13% of isolates. Against carbapenem-non-susceptible strains, % susceptibilities were: FTB, 68.5%, CZA, 63.0%, C/T, 59.3%; and MVB, 21.3% (Table 2).

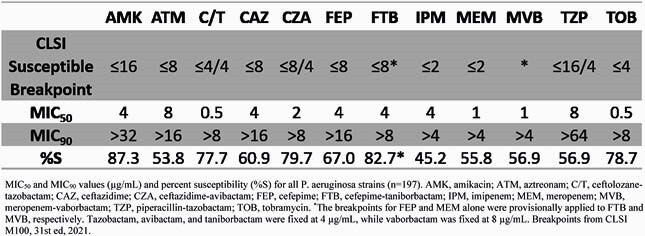

MIC50 and MIC90 values (µg/mL) and percent susceptibility (%S) for all P. aeruginosa strains (n=197). AMK, amikacin; ATM, aztreonam; C/T, ceftolozane-tazobactam; CAZ, ceftazidime; CZA, ceftazidime-avibactam; FEP, cefepime; FTB, cefepime-taniborbactam; IPM, imipenem; MEM, meropenem; MVB, meropenem-vaborbactam; TZP, piperacillin-tazobactam; TOB, tobramycin. *The breakpoints for FEP and MEM alone were provisionally applied to FTB and MVB, respectively. Tazobactam, avibactam, and taniborbactam were fixed at 4 µg/mL, while vaborbactam was fixed at 8 µg/mL. Breakpoints from CLSI M100, 31st ed, 2021.

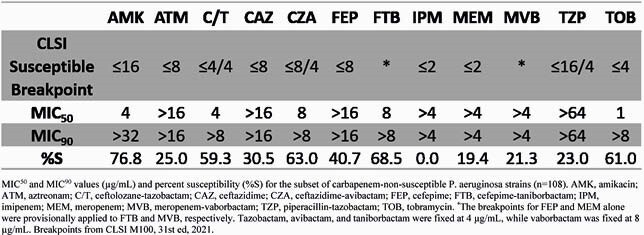

MIC50 and MIC90 values (µg/mL) and percent susceptibility (%S) for the subset of carbapenem-non-susceptible P. aeruginosa strains (n=108). AMK, amikacin; ATM, aztreonam; C/T, ceftolozane-tazobactam; CAZ, ceftazidime; CZA, ceftazidime-avibactam; FEP, cefepime; FTB, cefepime-taniborbactam; IPM, imipenem; MEM, meropenem; MVB, meropenem-vaborbactam; TZP, piperacillin-tazobactam; TOB, tobramycin. *The breakpoints for FEP and MEM alone were provisionally applied to FTB and MVB, respectively. Tazobactam, avibactam, and taniborbactam were fixed at 4 µg/mL, while vaborbactam was fixed at 8 µg/mL. Breakpoints from CLSI M100, 31st ed, 2021.

**Conclusion:**

Compared to MVB, CZA, and C/T, FTB demonstrated the greatest activity against the 197 *P. aeruginosa* strains tested, including many carbapenem-non-susceptible strains. Pending completion of clinical development, FTB may be a promising therapeutic option for MDR *P. aeruginosa* infections.

**Disclosures:**

**Robin Patel, MD**, **1928 Diagnostics** (Consultant)**BioFire Diagnostics** (Grant/Research Support)**ContraFect Corporation** (Grant/Research Support)**Curetis** (Consultant)**Hylomorph AG** (Grant/Research Support)**IDSA** (Other Financial or Material Support, Editor’s Stipend)**Infectious Diseases Board Review Course** (Other Financial or Material Support, Honoraria)**Mammoth Biosciences** (Consultant)**NBME** (Other Financial or Material Support, Honoraria)**Netflix** (Consultant)**Next Gen Diagnostics** (Consultant)**PathoQuest** (Consultant)**PhAST** (Consultant)**Qvella** (Consultant)**Samsung** (Other Financial or Material Support, Patent Royalties)**Selux Diagnostics** (Consultant)**Shionogi & Co., Ltd.** (Grant/Research Support)**Specific Technologies** (Consultant)**TenNor Therapeutics Limited** (Grant/Research Support)**Torus Biosystems** (Consultant)**Up-to-Date** (Other Financial or Material Support, Honoraria) **Robin Patel, MD**, BioFire (Individual(s) Involved: Self): Grant/Research Support; Contrafect (Individual(s) Involved: Self): Grant/Research Support; IDSA (Individual(s) Involved: Self): Editor’s stipend; NBME, Up-to-Date and the Infectious Diseases Board Review Course (Individual(s) Involved: Self): Honoraria; Netflix (Individual(s) Involved: Self): Consultant; TenNor Therapeutics Limited (Individual(s) Involved: Self): Grant/Research Support; to Curetis, Specific Technologies, Next Gen Diagnostics, PathoQuest, Selux Diagnostics, 1928 Diagnostics, PhAST, Torus Biosystems, Mammoth Biosciences and Qvella (Individual(s) Involved: Self): Consultant **David van Duin, MD, PhD**, **Entasis** (Advisor or Review Panel member)**genentech** (Advisor or Review Panel member)**Karius** (Advisor or Review Panel member)**Merck** (Grant/Research Support, Advisor or Review Panel member)**Pfizer** (Consultant, Advisor or Review Panel member)**Qpex** (Advisor or Review Panel member)**Shionogi** (Grant/Research Support, Scientific Research Study Investigator, Advisor or Review Panel member)**Utility** (Advisor or Review Panel member) **Vance G. Fowler, Jr., MD, MHS**, **Achaogen** (Consultant)**Advanced Liquid Logics** (Grant/Research Support)**Affinergy** (Consultant, Grant/Research Support)**Affinium** (Consultant)**Akagera** (Consultant)**Allergan** (Grant/Research Support)**Amphliphi Biosciences** (Consultant)**Aridis** (Consultant)**Armata** (Consultant)**Basilea** (Consultant, Grant/Research Support)**Bayer** (Consultant)**C3J** (Consultant)**Cerexa** (Consultant, Other Financial or Material Support, Educational fees)**Contrafect** (Consultant, Grant/Research Support)**Debiopharm** (Consultant, Other Financial or Material Support, Educational fees)**Destiny** (Consultant)**Durata** (Consultant, Other Financial or Material Support, educational fees)**Genentech** (Consultant, Grant/Research Support)**Green Cross** (Other Financial or Material Support, Educational fees)**Integrated Biotherapeutics** (Consultant)**Janssen** (Consultant, Grant/Research Support)**Karius** (Grant/Research Support)**Locus** (Grant/Research Support)**Medical Biosurfaces** (Grant/Research Support)**Medicines Co.** (Consultant)**MedImmune** (Consultant, Grant/Research Support)**Merck** (Grant/Research Support)**NIH** (Grant/Research Support)**Novadigm** (Consultant)**Novartis** (Consultant, Grant/Research Support)**Pfizer** (Grant/Research Support)**Regeneron** (Consultant, Grant/Research Support)**sepsis diagnostics** (Other Financial or Material Support, Pending patent for host gene expression signature diagnostic for sepsis.)**Tetraphase** (Consultant)**Theravance** (Consultant, Grant/Research Support, Other Financial or Material Support, Educational fees)**Trius** (Consultant)**UpToDate** (Other Financial or Material Support, Royalties)**Valanbio** (Consultant, Other Financial or Material Support, Stock options)**xBiotech** (Consultant) **Daniel D. Rhoads, MD**, **Becton, Dickinson and Company** (Grant/Research Support) **Michael Jacobs, MBBS**, **Venatorx Pharmaceuticals, Inc.** (Grant/Research Support) **Focco van den Akker, PhD**, **Venatorx Pharmaceuticals, Inc.** (Grant/Research Support) **David A. Six, PhD**, **Venatorx Pharmaceuticals, Inc.** (Employee) **Greg Moeck, PhD**, **Venatorx Pharmaceuticals, Inc.** (Employee) **Krisztina M. Papp-Wallace, Ph.D.**, **Merck & Co., Inc.** (Grant/Research Support)**Spero Therapeutics, Inc.** (Grant/Research Support)**Venatorx Pharmaceuticals, Inc.** (Grant/Research Support)**Wockhardt Ltd.** (Other Financial or Material Support, Research Collaborator) **Robert A. Bonomo, MD**, **entasis** (Research Grant or Support)**Merck** (Grant/Research Support)**NIH** (Grant/Research Support)**VA Merit Award** (Grant/Research Support)**VenatoRx** (Grant/Research Support)

